# Supplementary material for: Exposure to heavy metals, bisphenol A, and phthalates: Implications for precocious or delayed puberty
Source: PLoS One. 2025 Dec 31;20(12):e0322383. doi: 10.1371/journal.pone.0322383 (PMC12755744; doi:10.1371/journal.pone.0322383)
Supplement: S1 Table — (PDF) [file pone.0322383.s001.pdf]

S1 Table. Characteristics of study participants of the KorEHS-C (n=1,424)

**Variables and chemical levels**	**Precocious** (n = 50)	**Normal** (n = 1,320)	**Delayed** (n = 54)	**P**[a]
Age (years), mean (SD)	9.12 (3.15)	12.92 (3.65)	15.98 (1.38)	<0.001
Body mass index (kg/m^2), mean (SD)	18.93 (3.90)	19.81 (3.74)	21.24 (3.40)	0.005
Female sex (%)	38 (76.0%)	824 (62.4%)	12 (22.2%)	<0.001
Living in rural areas (%)	23 (46.0%)	873 (66.2%)	42 (77.8%)	0.011
Presence of sibling (%)	22 (44.0%)	581 (44.1%)	31 (57.4%)	0.154
At least one parent with college or university degree (%)	27 (55.1%)	675 (52.7%)	19 (38.8%)	0.148
Above-median household income[b]	10 (20.0%)	310 (23.5%)	12 (22.2%)	0.804
Blood and urinary measurement of heavy metals (geometric mean [SD])				
Lead, ug/dL	1.31 (1.48)	1.13 (1.46)	1.22 (1.52)	0.007
Mercury, ug/L	1.95 (1.54)	1.88 (1.51)	1.93 (1.57)	0.669
Cadmium, ug/L $_{creatinine}$	0.34 (1.62)	0.26 (1.65)	0.23 (1.63)	<0.001
Urinary measurement of BPA and phthalate metabolites (geometric mean [SD])				
BPA, ug/g $_{creatinine}$	2.03 (2.80)	1.00 (2.97)	0.94 (2.80)	<0.001
MBzP, ug/g $_{creatinine}$	9.87 (2.97)	5.10 (3.03)	5.93 (2.51)	<0.001
MECPP, ug/g $_{creatinine}$	59.74 (2.12)	33.78 (2.10)	25.53 (1.63)	<0.001
MnBP, ug/g $_{creatinine}$	66.02 (1.92)	42.95 (2.01)	34.12 (1.73)	<0.001
MEOHP, ug/g $_{creatinine}$	30.27 (2.46)	16.78 (2.23)	12.68 (1.65)	<0.001
MEHHP, ug/g $_{creatinine}$	40.85 (2.48)	23.1 (2.27)	17.46 (1.68)	<0.001

BPA; bisphenol A, DEHP; diethylhexyl phthalate, DBP; di-n-butyl phthalate, MEHHP; Mono-(2-ethyl-5-hydroxyhexyl) phthalate, MEOHP; Mono-(2-ethyl-5-oxohexyl) phthalate, MBP; Mono-N-Butyl phthalate.
