# Supplementary material for: Exposure to heavy metals, bisphenol A, and phthalates: Implications for precocious or delayed puberty
Source: PLoS One. 2025 Dec 31;20(12):e0322383. doi: 10.1371/journal.pone.0322383 (PMC12755744; doi:10.1371/journal.pone.0322383)
Supplement: S2 Table — (PDF) [file pone.0322383.s002.pdf]

S2 Table. Number of samples below the limit of detection of measurement

Chemicals	Study year / Sample	2012-2013			2014		
		LOD (ug/L)	No. of measurement	No. of <LOD	LOD (ug/L)	No. of measurement	No. of <LOD
Pb	Blood	0.189	1775	1	0.149	571	0
Hg	Blood	0.200	1775	0	0.121	571	0
Cd	Urine	0.071	1804	20	0.031	571	1
BPA	Urine	0.240	1803	122	0.51	571	2
MBzP	Urine	0.370	1800	11	0.27	571	0
MECPP	Urine	0.399	1800	2	0.34	571	0
MEHHP	Urine	0.473	1800	6	0.28	571	0
MEOHP	Urine	0.399	1800	5	0.26	571	0
MnBP	Urine	0.473	1800	1	0.44	571	0

LOD; limit of detection
