# Supplementary material for: Exposure to heavy metals, bisphenol A, and phthalates: Implications for precocious or delayed puberty
Source: PLoS One. 2025 Dec 31;20(12):e0322383. doi: 10.1371/journal.pone.0322383 (PMC12755744; doi:10.1371/journal.pone.0322383)
Supplement: S3 Fig — (PDF) [file pone.0322383.s003.pdf]

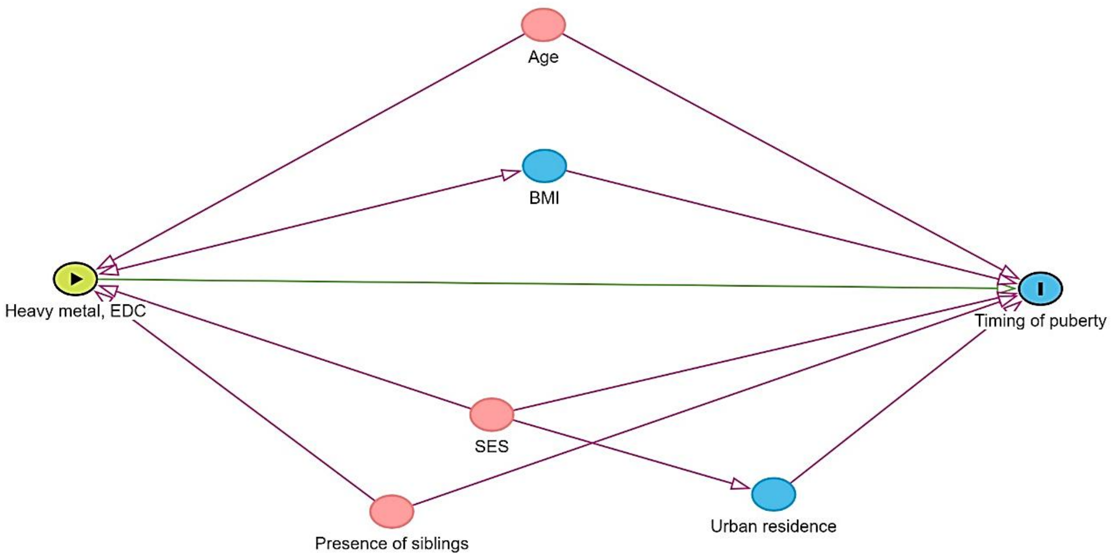

S3 Figure. Directed acyclic graph for the causal pathway. EDC, endocrine-disrupting chemicals; BMI, body mass index.
