# Supplementary material for: Exposure to heavy metals, bisphenol A, and phthalates: Implications for precocious or delayed puberty
Source: PLoS One. 2025 Dec 31;20(12):e0322383. doi: 10.1371/journal.pone.0322383 (PMC12755744; doi:10.1371/journal.pone.0322383)
Supplement: S5 Fig — (PDF) [file pone.0322383.s005.pdf]

<table>
<tr><td>A. Precocious puberty among those with low cadmium (ψ= 0.28, P=0.545)</td><td>B. Precocious puberty among those with high cadmium (ψ= -0.23, P=0.475)</td></tr>
<tr><td>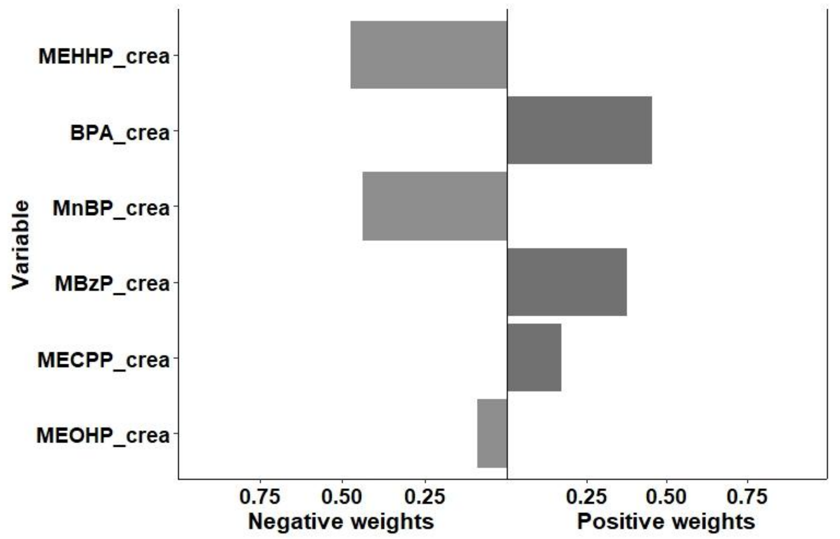
</td><td>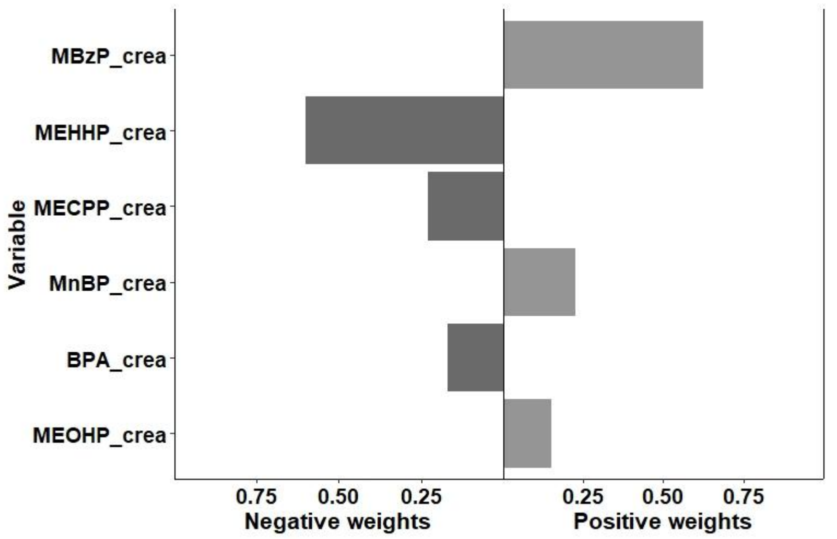
</td></tr>
<tr><td>C. Delayed puberty among those with low cadmium (ψ= 0.26, P=0.414)</td><td>D. Delayed puberty among those with high cadmium (ψ= 0.49, P=0.301)</td></tr>
</table>

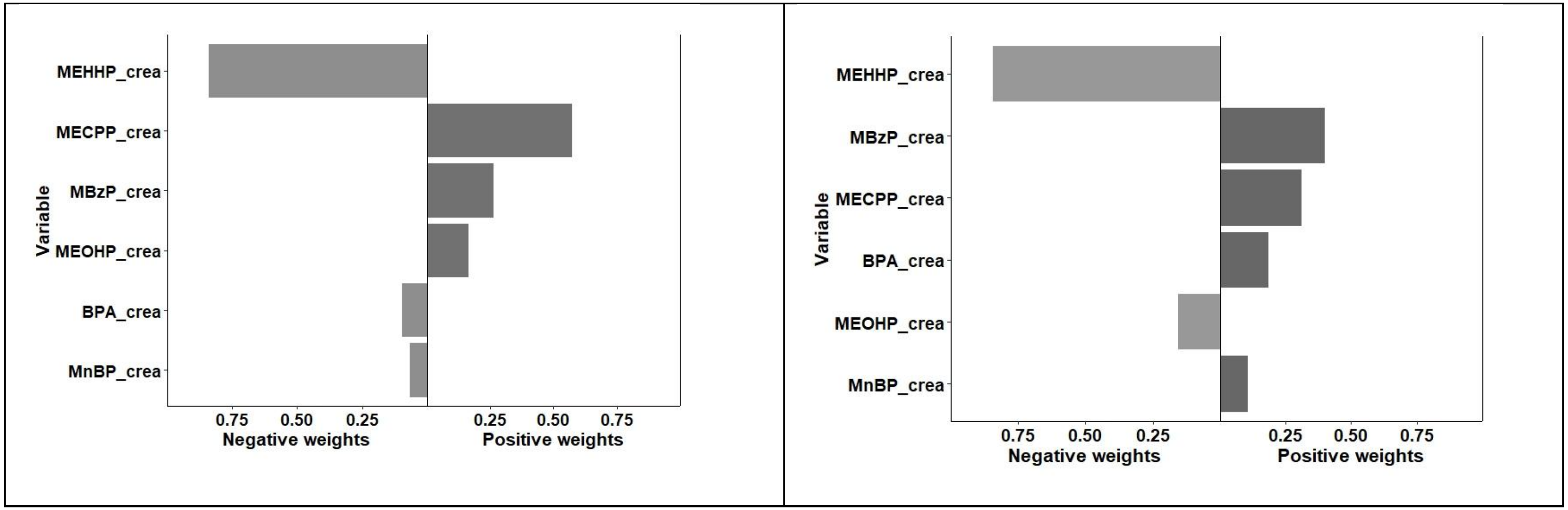

S5 Figure. Stratified association between mixture exposure to BPA, and phthalate metabolites and abnormal puberty.
