# Supplementary material for: Exposure to heavy metals, bisphenol A, and phthalates: Implications for precocious or delayed puberty
Source: PLoS One. 2025 Dec 31;20(12):e0322383. doi: 10.1371/journal.pone.0322383 (PMC12755744; doi:10.1371/journal.pone.0322383)
Supplement: Attachment — Submitted filename: PONE-D-25-12742_reviewer.docx [file pone.0322383.s006.docx]

The manuscript titled 'Exposure to heavy metals, bisphenol A, and phthalates: Implications for precocious or delayed puberty' is interesting and written well. I have only a few remarks:

Comment 1: In introduction, the author needs to put forward the hypothesis of this study and clarify the content and significance of his own research. In addition, What are the differences or innovations between this study and these findings?

Comment 2: In line 128-129, the authors need to add detailed test procedures or add references.

Comment 3: In table 1, need to label units, especially in Blood and urinary measurement of heavy metals, like table 2.

Comment 4: In results, it is suggested to add the corresponding figures, here are all tables. In addition, is there any correlation analysis that can be done between these data?

Comment 5: in discussion, it is suggested that the authors further enhance the depth of the discussion section of this paper.
