# Supplementary material for: Exposure to heavy metals, bisphenol A, and phthalates: Implications for precocious or delayed puberty
Source: PLoS One. 2025 Dec 31;20(12):e0322383. doi: 10.1371/journal.pone.0322383 (PMC12755744; doi:10.1371/journal.pone.0322383)
Supplement: Attachment — Submitted filename: Response to reviewer_R1.1.docx [file pone.0322383.s008.docx]

PONE-D-25-12742

Exposure to heavy metals, bisphenol A, and phthalates: Implications for precocious or delayed puberty

PLOS ONE

Dear Editor

Thank you so much for your reviews and comments which should much improved our manuscript quality. We responded each point raised.

\# Editorial comments

In the online submission form you indicate that your data is not available for proprietary reasons and have provided a contact point for accessing this data. Please note that your current contact point is a co-author on this manuscript. According to our Data Policy, the contact point must not be an author on the manuscript and must be an institutional contact, ideally not an individual. Please revise your data statement to a non-author institutional point of contact, such as a data access or ethics committee, and send this to us via return email. Please also include contact information for the third party organization, and please include the full citation of where the data can be found.

\# Response to editorial comments:

Thank you for your message and for reviewing our manuscript.

In accordance with the journal’s Data Policy, we have revised the data availability statement to designate a non-author, institutional point of contact. Please find the updated statement below:

The data are being reviewed and prepared for release, pending institutional approval. Currently data are available from the Environmental Health Research Department of National Institute of Environmental Research (<https://www.nier.go.kr>, contact point: [ojkim21@korea.kr](mailto:ojkim21@korea.kr)) for researchers who meet the criteria for access to confidential data.

Reviewers' comments:

Reviewer #1:

The manuscript titled 'Exposure to heavy metals, bisphenol A, and phthalates: Implications for precocious or delayed puberty' is interesting and written well. I have only a few remarks:

Comment 1: In introduction, the author needs to put forward the hypothesis of this study and clarify the content and significance of his own research. In addition, What are the differences or innovations between this study and these findings?

Response #1: We appreciate the reviewer’s comments. We have clarified our research hypothesis and implications of this study to the Introduction (Lines 60-64, p.4 in the revised manuscript).

Comment 2: In line 128-129, the authors need to add detailed test procedures or add references.

Response #2: We have added detailed procedure in Method (Lines 106-116, p.5-6)

Comment 3: In table 1, need to label units, especially in Blood and urinary measurement of heavy metals, like table 2.

Response #3: Table 1 was updated according to the reviewer’s comment. It is moved to S1 Table as the other reviewer’s suggestion (S1 Table).

Comment 4: In results, it is suggested to add the corresponding figures, here are all tables. In addition, is there any correlation analysis that can be done between these data?

Response #4: Fig 1 presents exposure profiles of urinary (A) cadmium, (B) BPA, (C) MECPP, (D) MnBP, (E) MEOHP, and (F) MEHHP levels in boys and girls with precocious, normal, and delayed puberty onset. We have added a correlation plot (S4 Figure) and corresponding descriptions (Line 189-190, p.9).

Comment 5: in discussion, it is suggested that the authors further enhance the depth of the discussion section of this paper.

Response #5: We elaborated more for the positive association between lead and delayed puberty (Lines 254-260, p.15).

Reviewer #2:

The manuscript has several major shortcomings that impact its overall scientific rigor.

Comment #1: The Abstract (lines 38–54) does not clearly highlight the cross-sectional nature, limiting causal interpretation.

Response #1: The Abstract was revised to explicitly state that our study is based on a cross-sectional design (Line 21, p.2).

Comment #2: Reliance on self-measured Tanner stage (line 43) risks misclassification, especially in younger participants.

Response #2: The assessment of pubertal timing relies on a combination of self-reporting and physical examination, which introduces the potential for measurement errors or misclassification of pubertal stages, especially in younger participants. However, given the prevalence of abnormal puberty timing is comparable to prior studies, we believe the potential measurement bias would have been minimal. We acknowledged this limitation in the Discussion (Lines 298-301, p.16).

Comment #3: Mixture analyses (lines 45–46) yield imprecise results (lines 52–53, 260), suggesting chance findings. Among the nine chemicals measured, only mono-benzyl phthalate (MBzP) showed a statistically significant association with delayed puberty in girls (lines 49–51, 259), while associations for other chemicals were weak and inconsistent, particularly in boys. The analysis lacks a detailed discussion of dose-response relationships, which is critical for establishing the biological plausibility of the reported associations. The paper reviews inconsistent findings from previous literature (lines 265–275) but does not adequately explain how the current study design resolves these inconsistencies or adds clarity.

Response #3: We acknowledge that our measurement of precocious puberty lacks the detailed timing information needed to support a dose–response analysis. This issue was addressed in Discussion (Lines 300-301, p.16).

Although the risk estimates were imprecise due to the rarity of events, effect sizes among girls were generally small—suggesting any association between chemical mixtures and abnormal puberty in this group is likely negligible. In boys, despite wide confidence intervals, the consistently positive direction of the estimates indicates a possible link between chemical exposure and abnormal puberty. We have addressed these limitations and their implications (Lines 306-310, p.16-17).

Our discussion was expanded to contrast our nationwide, biomarker-based design and mixture approach with prior small or single-chemical studies, clarifying how multi-exposure quantification enhances comparability (Lines 275-276, p.15).

Comment #4: Identification of lead’s key role (lines 276–278) not supported by sex-specific or stratified analyses.

Response #4: We clarified that the predominant positive weight of lead in the association between mixture exposure and delayed puberty is evidence only in boys (Line 252, p.14).

Comment #5: Only 50 precocious and 54 delayed cases (lines 47–48) may undermine power and model reliability.

Response #5: We have added a discussion of our limited power to detect modest mixture effects. Assuming a one-quartile increase in exposure confers a 1.5-fold higher risk of precocious puberty, our observed incidence yields only about 60% power. Nevertheless, as an exploratory study of environmental chemical mixtures, our results can guide future sample-size calculations needed to robustly detect such effects (Lines 285-292, p.16).

Reviewer #3:

Here is a list of specific comments. Note: line and page numbering in reviews and comments is based on ruler applied in Editorial Manager-generated PDF.

Comment #1. Page 3, line 48: There might be issues due to the small number of events per variable in the models, especially the multi-exposure models. The first limitation in the Discussion section addressed the low power issue. I suggest expanding the limitation to other issues such as overfitting, biased estimation, model instability, etc.

Response #1: We appreciate the reviewer’s comments. Assuming a one-quartile increase in exposure confers a 1.5-fold higher risk of precocious puberty, our observed incidence yields only about 60% power. Moreover, with few events relative to the number of exposure variables, there is an elevated risk of model overfitting, unstable parameter estimates, and biased odds ratios. Reliance on a single dataset may magnify these issues, underscoring the need for larger samples and independent replication in future studies to ensure robustness. Nevertheless, as an exploratory study of environmental chemical mixtures, our results can guide future sample-size calculations needed to robustly detect such effects (Lines 285-292, p.16).

Comment #2. Page 5, lines 91–92: I suggest reporting the number of participants excluded due to missing information on pubertal development, missing blood samples, and missing urine samples separately.

Response #2: We have added the number of participants excluded due to missing information on pubertal development, missing blood samples, and missing urine samples separately (Line 74, p.4). Our initial cohort included children aged 3–5 years, among whom 529 had missing data on pubertal development, and 42 did not provide biospecimens.

Comment #3. Page 7, lines 135–145: It might be worth considering two separate cohorts, one for the precocious puberty and the other for the delayed puberty. The precocious puberty cohort would consist of girls with age 10 years or younger and boys with age 8 or younger. The delayed puberty cohort would consist of girls with age 13 or older and boys with age 14 or older. In other words, Table S1 would be split into two tables.

Response #3: According to the reviewer’s suggestion, we corrected Table S1 stratified by precocious and delayed cohorts and sex, and then moved to new Table 1. Further, the old table 1 was moved to the S1 Table shown by category of precocious, normal, and delayed puberty.

Comment #4. Page 7, lines 148–150: I suggest rewriting the sentence as follows. ‘The selected covariates included participants’ age at the time of the tests, body mass index (BMI), presence of siblings, urban residence, and socioeconomic status (SES).’

Response #4: We updated the sentence just as suggested (Lines 136-138, p.6).

Comment #5. Page 7, line 150: BMI is a well-known metric and does not require a definition. If you would like to keep the sentence, I suggest revising the sentence as ‘BMI was calculated as weight in kilograms divided by height in meters squared.’

Response #5: Thanks to the reviewer’s suggestion, we removed the redundant sentence.

Comment #6. Page 7, lines 153–154: I suggest revising “the median” as ‘the median household income for 2014’ and removing the subsequent sentence, “the median household income for 2014 was . . . .” I suggest being specific about the median household income for 2014.

Response #6: We revised the sentence: “SES was defined by the higher educational attainment of either parent and by household income exceeding the 2014 median” (Line 140, p.6).

Comment #7. Page 7, lines 157–158: I suggest rewriting the sentence as follows. ‘Descriptive statistics summarized the baseline clinical characteristics of the study sample.’

Response #7: We updated the sentence as suggested (Lines 143, p.7).

Comment #8. Page 8, lines 158–159: I suggest rewriting the sentence as follows. ‘Due to their skewness, the urine levels of the measured environmental chemicals were log-transformed in regression models.’

Response #8: We revised the sentence as indicated (Lines 143-145, p.7).

Comment #9. Page 8, lines 159–160: I suggest stating the purpose of examining the correlations among the environmental chemicals.

Response #9: We clearly described the purpose of the test: “Spearman’s rank correlation was used to evaluate pairwise associations among the environmental chemicals and to identify strongly correlated compounds” (Lines 145-146, p.7).

Comment #10. Page 8, lines 161–162: I suggest revising the sentence as ‘each single-exposure model adjusted for age, BMI, presence of siblings, urban residence, and SES,’ and relocating the revised sentence to line 165 (after the next sentence).

Response #10: The sentence was revised following the suggestion (Lines 149-150. p.7).

Comment #11. Page 8, line 163: I suggest unifying the use of “environmental chemicals” and “heavy metals, BPA, or phthalate metabolites.”

Response #11: We updated the phrase accordingly (Line 153, p.7).

Comment #12. Page 8, line 166: The two blood chemicals, Pb and Hg, were not log-transformed. The phrase was not applicable to all chemicals.

Response #12: We apologize for the confusion: both blood measurements and all urine chemical concentrations were log-transformed for analysis: “Due to their skewness, the levels of all the measured environmental chemicals were log-transformed in regression models.” (Line 143-145. p.7).

Comment #13. Page 8, line 169: I suggest revising “all covariates” as ‘all covariates listed in the single-exposure models.’

Response #13: We updated the phrase as suggested (Line 154, p.7).

Comment #14. Page 8, lines 174–175: Please clarify if it was the one-quartile change in average chemical exposure or in average ‘log’ chemical exposure because the urine levels were log-transformed.

Response #14: The sentence was clarified: “The OR of each chemical mixture associated with a simultaneous one-quartile increase in the mean log-transformed exposure level was estimated” (Lines 162-163, p.7).

Comment #15. Page 8, line 180: There was no Table S3. Did you mean Table S1?

Response #15: Right. We corrected the typo and revised the paragraph according to our updated S1 Table (Line 169, p. 8).

Comment #16. Table 1: I suggest not reporting characteristics by the outcome status. I suggest replacing the current Table 1 with Table S1 (after revision).

Response #16: After corrections suggested in comment #3, we changed Table 1 with the revised S1 Table and description in the result (Table 1; Lines 16-173, p.8).

Comment #17. Figure 1: For the same reason as Comment 16 above, I suggest removing Figure 1.

Response #17: Figure 1 was removed in our revised manuscript as suggested.

Comment #18. Page 10, lines 209–210: Following Comment 9 above, what was the implication of these observed high correlations? If the purpose of reporting correlations was to avoid collinearity, these high correlations might distort the estimates of the multi-exposure models.

Response #18: Compared to most traditional multivariable regression models that suffer from collinearity issues when including multiple correlated variables, quantile g-computation can reduce the impact of extreme correlations between individual exposures by working with quantiles of mixtures rather than the original continuous values (Lines 157-160, p.7).

Comment #19. Table 2: I suggest providing a better indication of which exposures were log-transformed.

Response #19: We revised Table 2 indicating the chemical levels are log-transformed (Table 2).

Comment #20. Table S1: Different age categories in girls and boys made the distributions difficult to interpret. Please consider the two-cohort approach suggested in Comment 3 above.

Response #20: We presented summary statistics stratified by sex and cohorts (Table 1).

Comment #21. Figure S3: I suggest clarifying what EDC stands for.

Response #21: We have included a description of EDC in the figure legend (S3 Figure).
