# Supplementary material for: Exposure to heavy metals, bisphenol A, and phthalates: Implications for precocious or delayed puberty
Source: PLoS One. 2025 Dec 31;20(12):e0322383. doi: 10.1371/journal.pone.0322383 (PMC12755744; doi:10.1371/journal.pone.0322383)
Supplement: Attachment — Submitted filename: R2_Response letter_v2.docx [file pone.0322383.s010.docx]

PONE-D-25-12742R1

Exposure to heavy metals, bisphenol A, and phthalates: Implications for precocious or delayed puberty

PLOS ONE

Dear Editor

Thank you for reviewing our manuscript and for the constructive comments. Below we addressed each point. We will look forward to your feedback.

Major Concerns

Comment #1: Statistical power and model reliability: The number of cases of precocious puberty (50 cases) and delayed puberty (54 cases) is too low. Especially, the exposure model includes 9 chemicals, which may lead to overfitting of the model and unstable estimates (e.g., OR for boys with precocious puberty = 2.34, 95% CI: 0.36 - 15.14). The authors mentioned that the test power is only about 60% (assuming OR = 1.5), but did not fully discuss its potential impact on negative results (such as the possibility that no association with other chemicals may be due to insufficient power). I think the discussion section needs to be expanded to clearly explain the impact of small sample size on the mixed model (such as the risk of overfitting) and suggest the required sample size for future studies. Add sensitivity analysis and add model stability tests (such as Bootstrap method to verify the confidence interval of OR).

Response #1: We appreciate the editor’s comments. We have expanded the Discussion section to acknowledge the potential risk of overfitting and unstable estimates in our nine-chemical mixture model (Lines 301-317). However, given the limited research on the association between multiple chemical exposures and early puberty, our exploratory analysis may serve as a starting point to stimulate further investigation in this area (Lines 317-319, p.17).

Comment #2: Limitations of exposure assessment: Relying solely on a single biological sample (blood/urine) cannot reflect the dynamic of long-term exposure, especially for heavy metals with long half-lives (such as lead accumulation in bones). Other environmental pollutants (such as pesticides, flame retardants) were not included, which may miss important confounding factors. The authors need to strengthen the discussion and clearly state the limitations of single measurement in the "Limitations" section. It is recommended that future studies adopt a longitudinal design or cumulative exposure indicators. Supplementary analysis: If the data permit, analyze the interactions between chemical substances (such as heavy metals and phthalates).

Response #2: We clearly have addressed that single blood/urine measurements may not capture long‑term exposure and that additional pollutants were not assessed, potentially leaving residual confounding. We therefore recommended future longitudinal designs or cumulative exposure biomarkers.

To address the potential interaction by heavy metal exposure, we assessed stratified associations between mixture exposure to BPA, and phthalate metabolites and abnormal puberty by low (less than median) or high (median or higher) urinary cadmium level. The additional findings are described in our manuscript (Lines 164-167 & 226-228; S5 Figure).

Comment #3: Rigor of result interpretation: Only MBzP showed a significant effect on girls' delayed puberty (OR = 2.75), while the other associations were not significant or had wide confidence intervals. However, the conclusion that "chemical mixtures are associated with delayed puberty" may be an overinterpretation. The discussion on the mechanism of urban-rural differences (with a higher proportion of girls experiencing delayed puberty in rural areas) is insufficient (such as whether it is related to pollutant exposure levels or social economic factors?). Please revise the conclusion statement to avoid suggesting a "wide correlation of mixtures", and focus on the significance of MBzP and the potential role of lead in boys. Delve deeper into the differences between urban and rural areas, and supplement with literature support (such as citing Oh et al. 2024's discussion on air pollution and precocious puberty). Distinguish the effects of pollution exposure from those of confounding factors such as obesity.

Response #3: We have revised our discussion to focus on the robust MBzP association with girls’ delayed puberty and the suggestive lead effect in boys, removing any language implying a positive mixture effect (Line 241-243, p.14). The discussion in our revised manuscript explores urban–rural differences in more depth, with additional citation of similar findings (Line 287-289, p.15-16). According to our conceptual pathway explaining the association between chemical mixture exposure and puberty timing, urban residence and obesity could be separate confounders.

Comment #4: Methodological details and data accuracy: In Table 3, "MBaP" should be "MBzP" (Page 21). Inconsistent ethical approval numbers: The main text states 2013-05-004-001 (Page 12), but the supplementary material is 2013-05-004_(III).pdf (Page 38). The correlation coefficients are extremely high (such as MEOHP-MEHHP, ρ=0.97) and the impact of multicollinearity on the model has not been discussed. The authors should correct the data errors, unify the ethical approval numbers, and correct "MBaP" in Table 3. Provide an explanation for handling multicollinearity, clearly explain how QGC reduces the influence of multicollinearity (such as quantile transformation), or provide a VIF analysis in the supplementary material.

Response #4: We corrected typos in the manuscript. The ethical‑approval number is consistently listed as 2013‑05‑004‑001. You can check the same number at the ethical approval report by opening the file. However, the number shown in the file name in the supplementary data is missing the trailing "001". Compared to most traditional multivariable linear regression models that suffer from collinearity issues when including multiple correlated variables, QGC method can reduce the impact of extreme correlations between individual exposures by working with quantiles of mixtures rather than the original continuous values (Lines 156-158, p.7). In addition, QGC estimates a single overall effect (psi) for a simultaneous one-quantile increase in all exposures, rather than separate β-coefficients for each original exposure. Because we do not interpret or report separate parameter estimates that could be inflated by collinearity, the VIF is neither meaningful nor necessary (Keil AP, Buckley JP, O'Brien KM, Ferguson KK, Zhao S, White AJ. A Quantile-Based g-Computation Approach to Addressing the Effects of Exposure Mixtures. Environ Health Perspect. 2020;128(4):47004.).

Minor Concerns

Comment #1: Chart optimization: Table 1: Add units (such as blood lead μg/dL) and provide a footnote stating "median [IQR]" or "mean (SD)". Figure S4: Simplify the correlation coefficient graph, highlighting the high correlations with |ρ| > 0.7.

Response #1: In Table 1 we added units (e.g., blood lead μg/dL; urinary phthalates μg/g creatinine) and footnotes indicating “median [IQR]”(Table 1). Figure S4 has been revised to highlight only correlations |ρ|>0.7 (S4 Figure).

Comment #2: Discussion Extension: Analyze the potential mechanisms underlying the significant effect of MBzP in girls and the lack of such effect in boys (such as differences in sex hormone pathways). Refer to the latest literature to support the discussion on the urban-rural disparity.

Reponse #2: We added discussion of why the association between MBzP and abnormal puberty was not evident in boys of our study population. Because the positive association between phthalate exposure and delayed puberty was stronger in obese boys. This may explain the weaker association among the boys of our study population who show generally low BMI. We additionally cited a recent related literature (Lines 278-280, p.15).

Comment #3: Terminology Consistency: The term "phthalate metabolites" (instead of "phthalates") is uniformly used throughout the text to avoid confusion between the parent compound and its metabolites.

Response #3: We have reviewed the paper thoroughly and ensured all instances of “phthalates” mean the exposure, not the urinary biomarkers, to clarify we measured metabolites rather than parent compounds.
