# Supplementary material for: Exposure to heavy metals, bisphenol A, and phthalates: Implications for precocious or delayed puberty
Source: PLoS One. 2025 Dec 31;20(12):e0322383. doi: 10.1371/journal.pone.0322383 (PMC12755744; doi:10.1371/journal.pone.0322383)
Supplement: Attachment — Submitted filename: Comments.docx [file pone.0322383.s009.docx]

**Comment1:**

Effects of background factors and other issues were difficult to control even in single- and joint-exposure analyses performed in the current study. It’s another limitation which should be clarified. Actually there are so many limitations in the current study.

**Comment 2:**

"Our findings contribute to the growing body of evidence implicating environmental chemicals that have lifelong health impacts on pubertal development."…..was mentioned in the conclusion.

The current study did not focus on all the environmental chemicals so it should be recommended not to say environmental chemicals in the conclusion rather sharing in a more specific way regarding heavy metals and other chemicals included in the study. Longitudinal study should be needed to say the lifelong impact.
