# Supplementary material for: Exposure to heavy metals, bisphenol A, and phthalates: Implications for precocious or delayed puberty
Source: PLoS One. 2025 Dec 31;20(12):e0322383. doi: 10.1371/journal.pone.0322383 (PMC12755744; doi:10.1371/journal.pone.0322383)
Supplement: Attachment — Submitted filename: Rebuttal letter_R2.docx [file pone.0322383.s011.docx]

PONE-D-25-12742R2

Exposure to heavy metals, bisphenol A, and phthalates: Implications for precocious or delayed puberty

PLOS ONE

Dear Editor

We appreciate the reviewer’s comments. Please find our point-by-point responses and revised manuscript. We will look forward to your feedback.

Best regards,

**<Reviewer #4>**

Comment #1: The present study includes an overall adequate sample size and has applied sound analytical methods. However, the way results are presented, together with certain limitations—some of which were already acknowledged in the manuscript—makes the findings less interpretable and difficult to draw valid inferences from:

The sample size in the groups with precocious or delayed puberty is very small, especially when analyses are stratified by sex. There were 50 children with precocious puberty (38 girls and 12 boys) and 54 children with delayed puberty, of whom only 12 were girls. This limited subgroup size substantially reduces the robustness of the statistical analyses.

Response #1: We appreciate the reviewer’s comments. We agree that the subgroup sizes were small, especially for boys with precocious puberty and girls with delayed puberty. We have addressed an explicit acknowledgment of limited power (about 60%), and emphasized that the study is exploratory, aiming to provide effect-size estimates to inform future longitudinal or pooled analyses (Lines 300-301; 304-305, p.16; 324-326, p.17 in the revised manuscript).

Comment #2: Regarding the inclusion of “having siblings” in the DAG, the rationale for its relationship with the level of exposure to heavy metals, is unclear. Further clarification is needed on how this variable may confound or mediate the associations under study.

Response #2: Having siblings can reflect differences in household characteristics that may indirectly influence both exposure levels and developmental outcomes. Including this variable helps to partially control unmeasured household-level confounding (Lines 140-142, p.6-7).

Comment #3: Among boys, because pubertal status was defined based on the current questionnaire, boys aged 9–13 years were excluded from the study. However, a similar exclusion was not applied to the girls. The justification for this difference between sexes is not provided.

Response #3: The exclusion of boys aged 9–13 years was based on the definition of precocious and delayed puberty using Tanner stage criteria. While girls in this age group were eligible for precocious puberty identification, determining precocious or delayed puberty for boys aged 9–13 years was not possible based on the single measurement taken at the time of the survey. This issue was addressed in Method (Line 130-132, p.6).

Comment #4: The manuscript reports that 874 girls were included in the study, yet Table 1 indicates that 771 girls were included in the total cohort and 387 in the delayed puberty cohort. This discrepancy needs to be clarified.

Response #4: Thanks to the reviewer, we corrected the number in Table 1. The total number of girls included in the analysis of precocious puberty was 874 (Table 1).

Comment #5: In lines 184–188, the authors make comparisons that are statistically invalid. For example, they state that more girls were in the precocious puberty group (38 of 50). Since the number of boys and girls in the cohort is not equal, such a comparison is not appropriate. Instead, the correct approach would be to compare the proportion of children experiencing precocious puberty within each sex cohort (i.e., the percentage of girls versus the percentage of boys)

Response #5: We agree with the reviewer and have revised the text, presenting the percentage of girls versus the percentage of boys (Lines 189-191, p.9).

Comment #6: The definition of precocious and delayed puberty in your study is inherently age-dependent, as early puberty is only detectable in younger children and delayed puberty only in older children. Consequently, age is strongly collinear with the outcome. Entering age directly into the regression model may therefore lead to problems such as overadjustment or complete separation, making the estimates unstable or difficult to interpret. A more appropriate strategy would be to restrict the analysis to an age window where both exposed and unexposed children with and without the outcome are observed or..

Response #6: We have clarified that age was included as a covariate because it can act as a common cause influencing both chemical exposure and pubertal status. Excluding age from the model would have introduced residual confounding, although the potential multicollinearity between the covariates. Given that pubertal status was assessed using Tanner staging, it was not feasible to apply age-standardized measures in our study (Lines 139-140, p.6).

**<Reviewer #5>**

Comment #1: Effects of background factors and other issues were difficult to control even in single- and joint-exposure analyses performed in the current study. It’s another limitation which should be clarified. Actually there are so many limitations in the current study.

Response #1: We agree that unmeasured or residual confounding attributed to background factors is an important limitation. Unmeasured environmental, nutritional, or genetic factors may remain as a source of residual confounding effects. We have clarified these limitations in the revised manuscript to ensure balanced interpretation of our findings (Lines 306-310, p.16).

Comment #2: "Our findings contribute to the growing body of evidence implicating environmental chemicals that have lifelong health impacts on pubertal development."
The current study did not focus on all the environmental chemicals so it should be recommended not to say environmental chemicals rather sharing in a more specific way regarding heavy metals and other chemicals included in the study. Longitudinal study should be needed to say the lifelong impact.

Response #2: We appreciate the reviewer’s comment. The conclusion has been revised to specify the chemical classes studied: “Our findings contribute to the growing body of evidence implicating the potential role of heavy metals, BPA and phthalate metabolites in pubertal development.” (Lines 335-336, p.17).

**<Reviewer #6>**

Comment: The author has addressed the reviewers' comments, implemented appropriate revisions, and incorporated additional content that enhances the overall completeness and scholarly rigor of the manuscript. The study demonstrates a degree of innovation and holds academic significance within the relevant field. It is therefore recommended for acceptance.

Response: We appreciate the reviewer’s comment.

**<Reviewer #7>**

Using data from the Korea Environmental Exposure and Health Survey in Children and Adolescents, the authors conducted a cross-sectional study to look at the associations between exposure to heavy metals, bisphenol A, and phthalates and precocious or delayed puberty. The authors reported that a higher mono-benzyl phthalate concentration was associated with a higher risk of delayed puberty in girls in single-exposure models, concluding that exposure to environmental chemicals may contribute to delayed puberty.

Overall, if the research questions examined in this study were to be replicated in numerous robust studies, the findings could inform public health interventions aimed at reducing the impact of modifiable environmental pollutants on puberty.

With that in mind, this reviewer has the following to remark:

Comment #1: The authors have made substantial modifications to their manuscript, improving clarity and overall quality. This is evident from the detailed feedback provided by the editor and reviewers.

Response #1: We appreciate the reviewer’s comment.

Comment #2: However, a major concern with this study is statistical power. When interpreting the results, it’s crucial to assess whether statistical power was sufficient to detect at least moderate-effect sizes to answer the research questions raised.

While the authors, as recommended by the editor, have acknowledged the potential risks of overfitting and unstable estimates in their multi-exposure model, the paper could guide future studies on how to proceed. In their discussion section, the authors could suggest strategies, apart from increasing the sample size (which is a given), to improve the likelihood of detecting a true effect, if one exists.

I hope this review is helpful and wish the authors the very best with their research!

Response #2: We agree that the subgroup sizes were small, especially for boys with precocious puberty and girls with delayed puberty. We have explicitly acknowledged the limited power (60%) of our study in Discussion. We emphasized that the study is exploratory, aiming to provide effect-size estimates to inform future longitudinal or pooled analyses (Lines 300-301; 303-305, p.16).
